# Cellular Uptake of siRNA-Loaded Nanocarriers to Knockdown PD-L1: Strategies to Improve T-cell Functions

**DOI:** 10.3390/cells9092043

**Published:** 2020-09-07

**Authors:** Raweewan Thiramanas, Mengyi Li, Shuai Jiang, Katharina Landfester, Volker Mailänder

**Affiliations:** 1 Dermatology Clinic, University Medical Center of the Johannes Gutenberg-University Mainz, Langenbeckstr. 1, 55131 Mainz, Germany; raweewan@nanotec.or.th; 2 Max Planck Institute for Polymer Research, Ackermannweg 10, 55128 Mainz, Germany; limengyi@mpip-mainz.mpg.de (M.L.); jiangs@mpip-mainz.mpg.de (S.J.)

**Keywords:** siRNA, PD-L1, T-cells, adoptive immunotherapy, nanocarriers, cellular uptake

## Abstract

T-cells are a type of lymphocyte (a subtype of white blood cells) that play a central role in cell-mediated immunity. Currently, adoptive T-cell immunotherapy is being developed to destroy cancer cells. In this therapy, T-cells are harvested from a patient’s blood. After several weeks of growth in culture, tumor-specific T-cells can be reinfused into the same cancer patient. This technique has proved highly efficient in cancer treatment. However, there are several biological processes that can suppress the anti-cancer responses of T-cells, leading to a loss of their functionality and a reduction of their viability. Therefore, strategies are needed to improve T-cell survival and their functions. Here, a small interfering RNA (siRNA)-loaded nanocarrier was used to knockdown PD-L1, one of the most important proteins causing a loss in the functionality of T-cells. The biocompatibility and the cellular uptake of siRNA-loaded silica nanocapsules (SiNCs) were investigated in CD8^+^ T-cells. Then, the PD-L1 expression at protein and at mRNA levels of the treated cells were evaluated. Furthermore, the effect of the PD-L1 knockdown was observed in terms of cell proliferation and the expression of specific biomarkers CD25, CD69 and CD71, which are indicators of T-cell functions. The results suggest that this siRNA-loaded nanocarrier showed a significant potential in the delivery of siRNA into T-cells. This in turn resulted in enhanced T-cell survival by decreasing the expression of the inhibitory protein PD-L1. Such nanocarriers could, therefore, be applied in adoptive T-cell immunotherapy for the treatment of cancer.

## 1. Introduction

T-cells, or T-lymphocytes, are some of the most important cells in the immune system. These can be divided into two main subtypes, according to their surface markers, into CD4^+^ and CD8^+^ T-cells. CD4^+^ T-cells (T-helper cells) enhance the maturation of B-cells as well as the activation of CD8^+^ T-cells. CD8^+^ T-cells (cytotoxic T-cells) play a major role in destroying virus-infected cells and tumor cells [1,2,3]. Given this outstanding ability to kill cancer cells, adoptive T-cell immunotherapy for cancer treatment using CD8^+^ T-cells was employed and studied intensively for the past decade with promising results [4,5]. In the first step of this specific immunotherapy, CD8^+^ T-cells were collected from the patient’s blood. These were selected and activated while undergoing culture conditions, in order to enhance the number of specific T-cells and to make them strong enough to attack cancer cells before being reinfused into the patient’s body [6]. However, T-cells start to lose their viability and function after a long expansion and cultivation time, leading to a loss in their ability to effectively kill cancer cells [4,5,6,7]. The decrease in T-cell survival and killing ability is due to the natural mechanism of the immune system. When antigen presenting cells, for example, dendritic cells, produce a cancer antigen on their surface and then bind to a T-cell receptor, this results in T-cell activation, trafficking along the blood stream to the cancer site, recognition of cancer, and finally killing the cancerous cells. After this process, the number of specific T-cells decreases to ensure that they become inactive, thus avoiding subsequent chronic inflammation and autoimmune disease. There are several proteins involved in T-cell inactivation, which are called immune checkpoint proteins, such as cytotoxic T-lymphocyte antigen 4 (CTLA-4), programmed cell death-1 (PD-1), and their cognate ligands (PD-L1 and PD-L2) [8,9,10,11,12]. One of the well-known T-cell mechanisms of inhibition occurs through PD-1/PD-L1 interaction through the specific binding of the PD-1 receptor on the surface of T-cells with its ligand (PD-L1), on the surface of dendritic cells. Many researchers focused on the inhibition of PD-1 expression, aiming at improving T-cell function [12]. In fact, PD-L1 is also expressed on the surface of T-cells, which can cause PD-1/PD-L1 interaction within the T-cell population itself, which finally blocks T-cell activation [13]. To inhibit the immune checkpoint proteins PD-1 or PD-L1 expression, it is necessary to pay careful attention to systemic blocking, which can result in an undetermined effect of an over-immune reaction. PD-L1 is not only expressed on the surface of hematopoietic cells, but is also found on the surface of non-hematopoietic cells [14]. As a selective mechanism of inhibition, small interfering RNA (siRNA)-mediated silencing of PD-L1 was used and electroporated into tumor-specific CD4^+^ and CD8^+^ human T-cells [13]. The transfected T-cells successfully increased the interferon-γ production and antigen-specific cytotoxicity. These findings suggest that siRNA-mediated PD-L1 silencing is another possible approach towards inhibiting the immune checkpoint proteins, leading to an enhanced T-cell function. However, it is known that the electroporation technique is harmful to cells, due to electric shock, resulting in decreased cell viability after transfection [15]. Given the great advantages of nanotechnology, nanocarriers containing payloads such as drugs, dyes, and specific biomolecules were widely developed and used. Among the various kinds of nanocarriers available, silica-based nanocapsules exhibit a high-loading capacity, ease of synthesis and surface functionalization. Furthermore, silicon oxide was approved by the U.S. Food and Drug administration (USFDA) for use in medical care products. Silica-based materials are accepted both due to their biocompatibility as well as their stability [16,17].

Therefore, the aim of this study was to inhibit the PD-L1 expression on CD8^+^ T-cells by using silica core shell nanocapsules, as nanocarriers to carry siRNA specific to PD-L1 mRNA. The silica nanocapsules (SiNCs) encapsulated siRNA-Cy5 dye was applied to the cells to track the cell uptake through flow cytometry. This was further confirmed by confocal laser scanning microscopy. The knockdown efficiencies at protein and mRNA levels were evaluated and the effects of the PD-L1 knockdown were investigated in terms of cell proliferation and specific biomarker expression.

## 2. Materials and Methods

### 2.1. Materials

Tetraethoxysilane (TEOS, 98%), cetyltrimethylammonium chloride (CTMA-Cl, 99%), cyclohexane (>99%), ammonium hydroxide (30% in water), and 3-aminopropyltrimethoxysilane (APTES, >98%) were purchased from Alfa Aesar (Kandel, Germany). Lutensol AT50 (LUT, 99%) and polyglycerol polyricinoleate (PGPR, >99%) were supplied by BASF (Ludwigshafen, Germany). The oil soluble surfactant poly((ethylene-*co*-butylene)-*b*-(ethylene oxide)), P(E/B-*b*-EO), consisting of a poly(ethylene-*co*-butylene) block (*M_w_* = 3700 g·mol^−1^) and a poly(ethylene oxide) block (*M_w_* = 3600 g·mol^−1^) was synthesized, starting from ω-hydroxypoly-(ethylene-*co*-butylene), which was dissolved in toluene, after addition of ethylene oxide via anionic polymerization [18]. All other chemicals were purchased from Sigma-Aldrich (Merck KGaA, Darmstadt, Germany) and used as received.

All siRNAs and primers were purchased from Sigma Aldrich (Merck KGaA, Darmstadt, Germany). The sequences were as follows: PD-L1#3 siRNAs (sense: 5′-GGAUAAGAACAUUAUUCAA[dT][dT]-3′, antisense: 5′-UUGAAUAAUGUUCUUAUCC[dT][dT]-3′), PD-L1#6 siRNAs (sense: 5′-CAUACAGCUGAAUUGGUCA[dT][dT]-3′, antisense: 5′-UGACCAAUUCAGCUGUAUG [dT][dT]-3′), scrambled PD-L1#3 siRNAs (sense: 5′-GAAACUCGUUAGAAAUUAA [dT][dT]-3′, antisense: 5′-UUAAUUUCUAACGAGUUUC[dT][dT]-3′), PD-L1 primers (forward: 5′-TATGGTGGTGCCGACTACAA-3′, reverse: 5′-TGCTTGTCCAGATGACTTCG-3′) and actin primers (forward: 5′-TTGCCGACAGGATGCAGAA-3′, reverse: 5′-GCCGATCCACACGGAGTACT-3′). MISSION^®^ siRNA Universal Negative Control #1 was used as the siRNA control. All antibodies were purchased from BioLegend (San Diego, CA, USA): anti-CD274-APC (anti-PD-L1, clone 29E.2A3), anti-CD25-FITC (clone BC96,) anti-CD69-PE (clone FN50), and anti-CD71-APC (clone CY1G4).

### 2.2. Synthesis of Silica Nanocapsules

The silica nanocapsules were synthesized in a water-in-oil mini-emulsion. For NC-1, 7 mg of NaCl and 20 µL of 25 wt% CTMA-Cl aqueous solution were dissolved in 0.5 mL of milli-Q water at the dispersed (water) phase. For the continuous (oil) phase, 5 mg of dodecylamine and 35 mg of P(E/B-*b*-EO) were dissolved in 7.5 mL of cyclohexane, and the mixture was added during the aqueous phase, while stirring at 500 rpm for 10 min at room temperature. Then, the emulsion was sonicated under ice cooling conditions for 180 s, at 70% amplitude in a pulse regime (20 s sonication, 10 s pause) using a Branson 450 W sonifier and a 1/2′′ tip. Afterwards, a cyclohexane solution containing 10 mg of P(E/B-*b*-EO) and 250 µL TEOS in 5 mL of cyclohexane was added dropwise to the mini-emulsion C, over a period of 20 min. The mixture was stirred at 25 °C for 24 h. For NC-2, an aqueous solution (0.5 mL) with pH 10.3 tuned by adding ammonia and containing 0.5 mg/mL of NaCl was used as the dispersed phase. A cyclohexane solution (6 mL) containing 160 mg PGPR surfactant was added to the water phase, while stirring at 500 rpm for 10 min. The subsequent sonication process for NC-2 was the same as that used for NC-1.

The synthesized nanocapsules were purified through repetitive centrifugation (3 times for 20 min, RCF 1664) and redispersion in cyclohexane, to remove the excess amounts of surfactant. When transferring the nanocapsules into aqueous media, 600 µL of nanocapsule dispersion in cyclohexane were added dropwise to 5 mL of Lutensol AT50 aqueous solution (1 wt%), while mechanically stirring, and the samples were then placed in an ultrasound bath for 3 min at 25 °C (25 kHz). Subsequently, the samples were stirred in open air at 25 °C, for 24 h, to allow the cyclohexane to evaporate.

For the encapsulation of Oligo Cy5 and siRNA in silica nanocapsules, 100 μL Oligo Cy5 solution (0.1 nmol/µL in water), or 100 μL siRNA solution (0.1 nmol/µL in water) were added during the water phase. The amount of water was reduced to maintain 500 μL of total volume during the water phase. All glassware, caps, spatula, and ultrasonication tip were rinsed with 70% EtOH. Sterile water, pipette tips, syringes, and tubes were used. The preparation process was performed on the ultra-clean bench, except for the centrifugation and reaction process.

### 2.3. Characterization of Silica Nanocapsules

The hydrodynamic diameter of silica nanocapsules was measured by using dynamic light scattering (DLS) with a Nicomp particle sizer (Model 380, PSS, Santa Barbara, CA, USA), at a fixed scattering angle of 90°. Zeta potential measurements were performed in 10^−3^ M potassium chloride solution at 25 °C, with a Malvern Zeta sizer (Malvern Instruments, Malvern, UK). The average results from three measurements were reported. The morphology of the nanocapsules was examined with a Jeol 1400 (Jeol Ltd., Tokyo, Japan) transmission electron microscope, operating at an accelerating voltage of 120 kV. TEM samples of nanocapsules were prepared by casting the diluted dispersions on carbon layer-coated copper grids.

### 2.4. Cell Culture

Human blood was taken from healthy donors at the Department of Transfusion Medicine, Mainz, after physical examination and after obtaining written informed consent, in accordance with the Declaration of Helsinki. The study was approved by the local Jurkat committee “Landesärztekammer Rheinland-Pfalz” (Bearbeitungsnummer: 837.439.12 (8540-F)). Peripheral blood mononuclear cells (PBMCs) were collected from the blood of healthy donors, by using the lymphocyte separation medium (Histopaque^®^-1077, Sigma-Aldrich, St. Louis, MO, USA). All experiments were performed in compliance with the relevant laws and institutional guidelines. The institutional ethics committee approved the study (Landesärztekammer Rheinland-Pfalz, 837.439.12 (8540-F)). Written informed consent was obtained for any experimentation we carried out when using samples from human subjects. As mentioned above, the studies were conducted in full accordance with the Declaration of Helsinki. Human PBMCs were co-stimulated with immobilized 0.1 µg/mL of anti-human CD3 (Clone OKT3, functional grade, eBioscience, Frankfurt am Main, Germany) and 100 U/mL recombinant human IL-2 (Novartis, Basel, Switzerland) at the Roswell Park Memorial Institute (RPMI, Gibco, New York, NY, USA); complete medium containing 10% fetal bovine serum (FBS, Gibco), 1% l-Glutamine (Gibco) and 1% penicillin/streptomycin (Gibco), according to the T-cell activation, in vitro protocol from eBioscience. After formation of cell aggregation at the bottom of the flask, CD8^+^ T-cells were harvested from the culture by a CD8 MicroBead and separated through the immunomagnetically MACS separation column (Miltenyi Biotec, Bergisch Gladbach, Germany). CD8^+^ T-cells were cultured in an RPMI complete medium and re-stimulated with anti-CD3 and IL-2, as mentioned above and incubated at 37 °C in a CO_2_-incubator, with 95% humidity and 5% CO_2_ (C200, Labotect, Rosdorf, Germany). The cell pellet was collected through centrifugation at 500 g for 5 min, resuspended in a RPMI medium and used for further assays. Viable cells were determined by the trypan blue exclusion method and counted by using the TC10™ automated cell counter (Bio-Rad, Hercules, CA, USA).

### 2.5. Cell Viability and Cell Proliferation Assay

To determine the half maximal effective concentration (EC_50_) of SiNCs, CD8^+^ T-cells resuspended in a RPMI medium containing 1% FBS and 100 U/mL IL-2 without antibiotics were seeded at a density of 20,000 cells per well in 0.1 µg/mL of an anti-CD3 pre-coated 96-well plate. Then, the cells were treated with various concentrations of SiNCs from 40–640 µg/mL for 24 h. A sample without any treatment was used as a negative control and calculated at 100% cell viability, while a 5% DMSO added sample was used as a positive control. After this, cell viability was evaluated by using the CellTiter-Glo^®^ luminescent cell viability assay (Promega, Fitchburg, MA, USA), according to the manufacturer’s protocol. This assay was based on the amount of ATP present, which reflected the presence of metabolically active cells. Luminescence was recorded 10 min after reagent addition, using a plate reader (Infinite^®^ M1000, Tecan, Männedorf, Switzerland). The EC_50_ values were calculated by fitting a curve with non-linear regression, using the GraphPad Program.

For the cell proliferation assay, CD8^+^ T-cells treated with SiNCs containing target siRNA or control siRNA, were compared to the cell control, to follow the proliferation of cells on day 2, using the same protocol as described above. A sample without any treatment was used as a control and calculated to be 100% cell proliferation.

### 2.6. Cellular Uptake Study by Flow Cytometry

For the cellular uptake study, CD8^+^ T-cells resuspended in an RPMI medium containing 1% FBS and 100 U/mL IL-2 without antibiotics were seeded at a density of 200,000 cells per well in 0.1 µg/mL of an anti-CD3 pre-coated 24-well plate. Then, the cells were treated with various concentrations of SiNCs for 24 h. Thereafter, the cells were collected from the plate, washed, resuspended in PBS, and stained with Zombie Aqua™ Fixable Viability Kit (BioLegend), according to the manufacturer’s instructions for live cell gating. Flow cytometry measurements were performed on an Attune™ NxT Flow Cytometer (Invitrogen, Waltham, MA, USA). Zombie Aqua™ dye was excited by the violet laser (405 nm) and had a maximum emission of 516 nm, which could be detected in channel VL-2. Cy5 positive-cells represented the cells that had taken up the SiNCs containing Cy5 dye and were recorded in channel RL-1 with the excitation of red laser (638 nm). Data analysis was performed using Attune™ NxT software (Invitrogen), by selecting the cells on a forward/sideward scatter plot, thereby excluding cell debris. These gated events were recorded by the histogram of fluorescent signal. Percentages of cell viability were recorded from living cells (Zombie Aqua negative-cells) compared to dead cells (Zombie Aqua positive-cells). After gating for living cells, percentages of Cy5 positive-cells were reported.

### 2.7. Cell Imaging by Confocal Laser Scanning Microscopy (cLSM)

For the confirmation of cellular uptake, CD8^+^ T-cells resuspended in an RPMI medium containing 1% FBS and 100 U/mL IL-2 without antibiotics were seeded at a density of 200,000 cells per well in 0.1 µg/mL of an anti-CD3 pre-coated 24-well plate. Then, the cells were treated with 80 µg/mL of SiNCs for 24 h. Following this, the cells were collected from the plate, washed, and transferred to a µ-Slide 8 well, with a glass coverslip bottom (Ibidi, Gräfelfing, Germany). Live cell images were taken using a commercial setup (LSM SP5 STED Leica Laser Scanning Confocal Microscope, Leica, Wetzlar, Germany), consisting of an inverse fluorescence microscope DMI 6000 CS, equipped with a multi-laser combination, five detectors operating in the range of 400–800 nm. A HCX PL APO CS 63 x 1.4 oil objective was used in this study. The excitation and detection conditions in a sequential mode could be described as follows. Fluorescent nanocapsules containing either the Oligo Cy5 or siRNA Cy5 dye were excited with a HeNe laser (633 nm), detected at 650–710 nm and pseudo-colored in green. The cell membrane was stained with CellMask™Orange (5 µg/mL, Life technologies, New York, NY, USA), excited with a DPSS laser (561 nm), detected at 570–600 nm, and pseudo-colored in red.

A co-localization assay was also performed to provide more evidence for cellular uptake. SiNCs were added to CD8^+^ T-cells, as mentioned above. Then, the cells were collected from the plate, washed, and transferred to a µ–Slide 8 well, with a glass coverslip bottom (Ibidi). Lysosome was stained with LysoTracker^®^ Green DND-26 (50 nM diluted in DMEM, Life Technologies, New York, NY, USA) for 1 h, excited with an Ar laser (488 nm), detected at 510–540 nm and pseudo-colored in (red). The membrane was stained with CellMask™Orange and pseudo-colored in blue. The SiNC was labeled with siRNA Cy5 and pseudo-colored in green. The merged images of three channels demonstrated that SiNCs encapsulated siRNA Cy5 were co-localized with lysosomes, as indicated by the white arrows.

### 2.8. PD-L1 Knockdown Study by Flow Cytometry

For the PD-L1 knockdown study, CD8^+^ T-cells resuspended in RPMI medium containing 1% FBS and 100 U/mL IL-2 without antibiotics were seeded at a density of 200,000 cells per well, in 0.1 µg/mL of an anti-CD3 pre-coated 24-well plate. Then, the cells were treated with SiNCs encapsulated with target siRNA that was specific to PD-L1 mRNA or SiNCs encapsulated with control siRNA that had no specific sequences to human mRNA compared to the cell control (without any capsule). After 24 h, FBS was added to the culture to obtain a final concentration at 20%. The cells were collected from the plate on a daily basis, washed, and stained with Zombie Aqua™ for live cell gating and anti-CD274-APC, or anti-CD25-FITC, or anti-CD69-PE, or anti-CD71-APC antibodies at 4 °C for 30 min. Subsequently, the cells were washed, resuspended in PBS, and analyzed using a flow cytometer, as described above. After gating for living cells, their median fluorescence intensities were used to calculate the relative expression of each cell surface marker.

### 2.9. Quantitative Polymerase Chain Reaction (qPCR) Analysis

After two days of treating the cells with SiNCs containing siRNA as described earlier, the cells were collected and the total RNA was extracted using the RNeasy Mini Kit (Qiagen, Germantown, MD, USA). The RNA concentration of the extracted RNA samples was measured using a Nanodrop (NanoDrop™ 8000 Spectrophotometer, Thermo Fisher Scientific, Wilmington, NC, USA). RT–PCR grade water (Invitrogen) was used throughout the experiment. Then, the cDNA was synthesized using the iScript™ cDNA Synthesis Kit (Bio-Rad) and the qPCR reaction was performed using the iQ™ SYBR^®^ Green Supermix (Bio-Rad) in a Thermal cycler (C1000, CFX96 Real-Time PCR detection system, Bio-Rad). The qPCR data was analyzed using the 2^−^*^ΔΔ^*^CT^ method. The results were expressed as a relative expression of two independent experiments, compared to the results of the control cell.

### 2.10. Statistical Analysis

The statistical significance was calculated by a *t*-test from duplicates, from at least two independent experiments. * *p* < 0.05, ** *p* < 0.01. *** *p* < 0.001.

## 3. Results 

In order to select an efficient nanocarrier that is able to deliver highly sensitive biomolecules such as siRNA into T-cells, Cy5 labeled oligonucleotides (oligo Cy5) were used to be encapsulated in various kinds of silica nanocapsules (SiNCs), as listed in Table 1, for the simulation of siRNA and the tracking of the capsules inside the cells. CD8^+^ T-cells stimulated with anti-CD3 antibody and IL-2 were treated with NC1 and NC2, using different surfactants. The capsules were stabilized with cationic surfactant cetyltrimethylammonium chloride (CTAC) and non-ionic surfactant Lutensol AT50 (LUT), to prevent aggregation, increase stability, and display different surface charges. The charges of the capsules were determined by the zeta-potential measurements; CTAC-stabilized capsules were slightly positive, while the LUT stabilized capsules were close to neutral.

**Figure 1:**
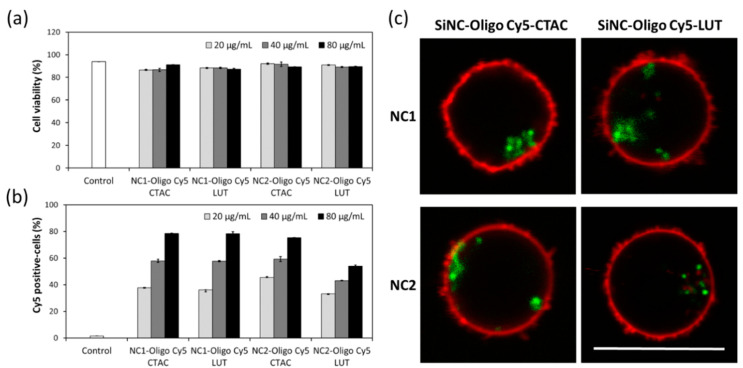
Cellular uptake study of SiNCs encapsulated Oligo Cy5 in CD8^+^ T-cells demonstrating—(**a**) Cell viability, (**b**) Cy5 positive-cells, and (**c**) confocal laser scanning microscopy (cLSM) images of CD8^+^ T-cells after treatment with SiNCs encapsulated Oligo Cy5 for 17 h. The membrane was stained with CellMask™Orange (red). The SiNC was labeled with Oligo Cy5 (green). The scale bars represent 10 μm.

The cellular uptake of SiNCs encapsulated oligo-Cy5 in CD8^+^ T-cells was studied in terms of toxicity and uptake, after 24 h of incubation. Flow cytometry revealed a slight decrease in cell viability of the CD8^+^ T-cells treated with SiNCs up to 80 µg/mL, indicating their biocompatibility with CD8^+^ T-cells (Figure 1a), and an increase of the Cy5 positive-cells with the increasing concentration of SiNCs (Figure 1b). The cellular uptake of these SiNCs was further confirmed by confocal laser scanning microscopy (cLSM) (Figure 1c). cLSM images showed green dots, representing SiNC-oligo Cy5 in the cytoplasm and indicating an effective uptake by the cells. In the experiments mentioned above, all SiNCs exhibited a good potential for the use as a nanocarrier to deliver biomolecules into CD8^+^ T-cells. However, the capsule NC2 showed a better capsule formation, whereby the shell structure was formed clearly, as observed in the TEM images (Supplementary Materials, Figure S1). Therefore, NC2 was chosen for use in subsequent experiments.

**Figure 2:**
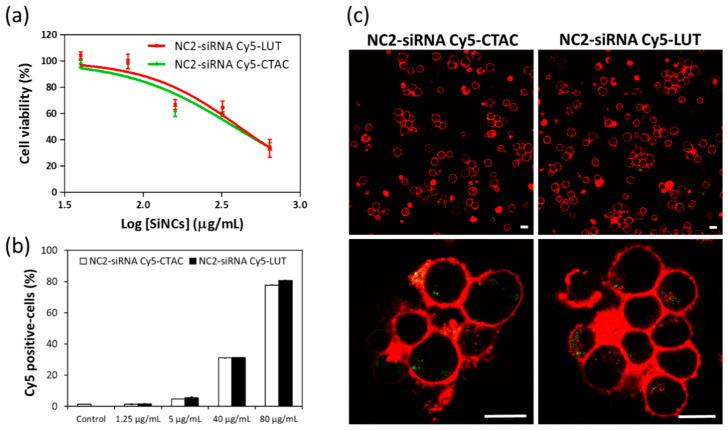
Cellular uptake study of SiNCs encapsulated siRNA Cy5 in CD8^+^ T-cells. (**a**) Cell viability, (**b**) Cy5 positive-cells, and (**c**) cLSM images of CD8^+^ T-cells, after treatment with SiNCs encapsulated siRNA Cy5 for 17 h. The membrane was stained with CellMask™Orange (red). The SiNC was labeled with Oligo Cy5 (green). The scale bars represent 10 μm. The EC_50_ of NC2-siRNA Cy5-CTAC and –LUT were 380 and 406 μg/mL, respectively. The scale bars represent 10 μm.

**Figure 3:**
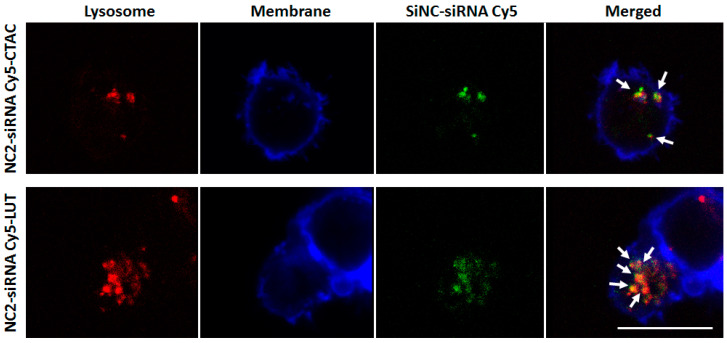
cLSM images of SiNCs-encapsulated siRNA Cy5, co-localized with lysosomes in CD8^+^ T-cells after 17 h. A co-localization assay was performed by staining lysosomes with LysoTracker dye (red). The membrane was stained with CellMask™Orange (blue). The SiNC was labeled with siRNA Cy5 (green). The merged images demonstrated that the SiNCs encapsulated siRNA Cy5 were co-localized with lysosomes, as indicated by the white arrows. The scale bars represent 10 μm.

To investigate the effect of different surface charges on cellular uptake, anti-CD3 antibody and IL-2 stimulated CD8^+^ T-cells were incubated with NC2-CTAC or -LUT encapsulated siRNA Cy5 for 24 h. The fluorescence-tracking molecule was changed from oligo-Cy5 to siRNA-Cy5 to closely imitate the real payload in this study, i.e., the siRNA that aimed at encapsulation inside the capsules and then transportation to the T-cells. Concentrations of SiNCs from 40 to 640 µg/mL were added to the cells to determine the critical toxic concentration. Figure 2a shows the increase of toxicity to CD8^+^ T-cells in a dose-dependent manner for both NC2-siRNA Cy5-CTAC and NC2-siRNA Cy5-LUT. The half maximum effective concentration (EC_50_) of NC2-siRNA Cy5-CTAC on CD8^+^ T-cells was 380 µg/mL, which was lower than that of NC2-siRNA Cy5-LUT (406 µg/mL). This result suggested that the LUT stabilized NC2 was more biocompatible with CD8^+^ T-cells than the CTAC stabilized NC2. The cellular uptake of these SiNCs was determined by an increase of the Cy5 positive-cells with an increasing concentration of SiNCs, using flow cytometry (Figure 2b), and was subsequently confirmed by cLSM (Figure 2c). When observing the percentages of Cy5 positive-cells, there was no significant difference between the CTAC and LUT-stabilized NC2, indicating that these were comparable in cellular uptake efficiency. It seemed that the charge of the capsule was not the only factor involved in cellular uptake efficiency, but other parameters also played a role, such as the components of capsule formation. cLSM images demonstrated that the CD8^+^ T-cells formed clumps, or were found to aggregate, with green dots representing NC2-siRNA Cy5 inside the cytoplasm. This result not only proved an efficient uptake by the cells, but also indicated the healthy or proliferation stage of the cells, due to the excellent biocompatibility of the capsules. To provide more evidence for cellular uptake, a co-localization assay was performed. Figure 3 shows that the NC2-siRNA Cy5-CTAC and -LUT were co-localized with lysosome, as indicated by the white arrows. This evidence provided solid proof of cellular uptake for these SiNCs in CD8^+^ T-cells. Although the EC_50_ did not show a significant difference, the cell morphology from the cLSM image of LUT-stabilized SiNC showed more cell clusters, indicating more healthy cells. Then, NC2-LUT was selected for use in the next steps, for the encapsulation of the control siRNA, which had no specific sequences in common with human mRNA and the target siRNA that contained specific sequences to PD-L1 mRNA.

**Figure 4:**
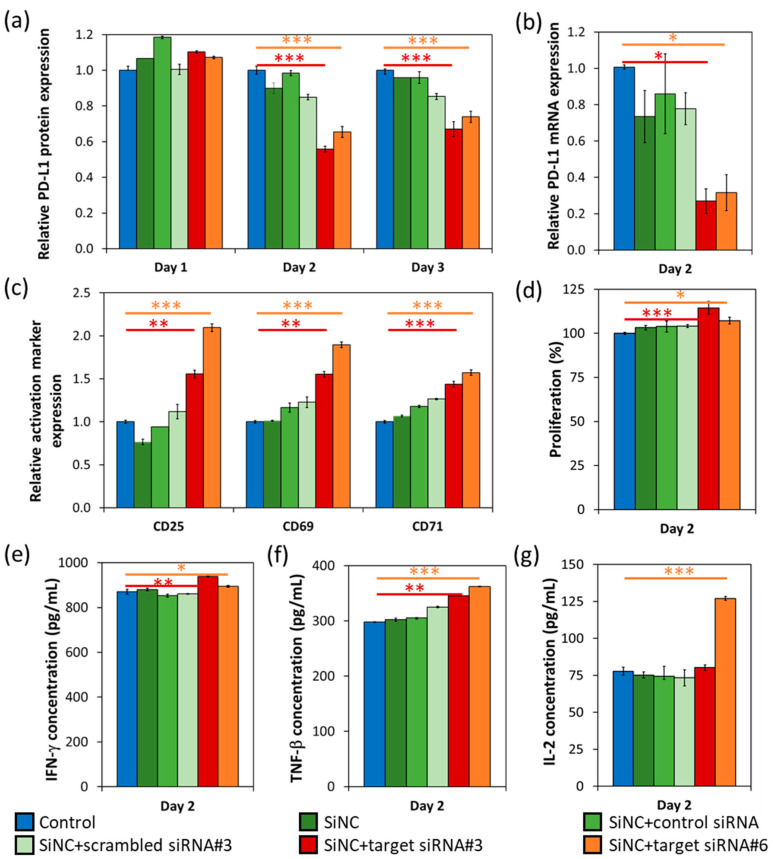
PD-L1 expression profiles and the effect of PD-L1 knockdown after use on the treated CD8^+^ T-cells with SiNC encapsulated target siRNA that are specific to PD-L1, compared to control (cells without any capsules), and those cells treated with SiNC, SiNC-encapsulated control siRNA, SiNC-encapsulated scrambled siRNA. Relative expression of (**a**) PD-L1 protein expression from day 1 to day 3, and (**b**) PD-L1 mRNA expression on day 2. The effect of PD-L1 knockdown was investigated on day 2. (**c**) Relative expression of CD25, CD69, and CD71. (**d**) Cell proliferation assay. ELISA assay of (**e**) IFN-γ, (**f**) TNF-β, and (**g**) IL-2 secretion. Statistical significance was calculated by *t*-test. * *p* < 0.05, ** *p* < 0.01. *** *p* < 0.001.

In this study, the target siRNAs specific to PD-L1 mRNA were preliminarily screened for their knockdown efficiency (data not shown). Target siRNA#3 and #6 were then selected and encapsulated in NC2-LUT (SiNC + target siRNA#3 or #6), which were subsequently added to the CD8^+^ T-cells. The PD-L1 knockdown efficiency and their knockdown effects were analyzed and compared by using four different controls—(1) cells only without any capsule (control), (2) bare NC2-LUT (SiNC), (3) NC2-LUT encapsulated with control siRNA, which has no specific sequence in common with human mRNA (SiNC + control siRNA), and (4) NC2-LUT encapsulated with scrambled siRNA, which has scrambled sequences to the target siRNA#3 (SiNC + scrambled siRNA#3), as shown in Figure 4. The PD-L1 knockdown efficiency at the protein level was determined from the relative PD-L1 protein expression profiles that were observed from day 1 to day 3, by flow cytometry analysis (Figure 4a). A decrease in PD-L1 protein production of T-cells treated with SiNC + target siRNA#3 and #6 compared to cell control was observed since day 2, with a knockdown efficiency of 44% and 35%; and an efficiency of 33% and 26% on day 3, respectively. Corresponding to the reduction of mRNA production analyzed by quantitative PCR (Figure 4b), the PD-L1 mRNA expression of T-cells treated with SiNC + target siRNA#3 and #6 dropped dramatically, when compared to the cell control with a knockdown efficiency at an mRNA level of 73% and 68% on day 2, respectively. The observation that the knockdown efficiency at mRNA level was higher than that at the protein level was quite common and these results were also found in other reports [19,20]. As the siRNA binds directly to the specific mRNA, it results in the degradation of the corresponding mRNA. Whereas the protein level not only depends on the mRNA synthesis, but also on the protein-half life and the translational steps [19]. The other controls (SiNC, SiNC + control siRNA, and SiNC + scrambled siRNA#3) all showed a slight reduction of the PD-L1 protein and the mRNA expression, compared to the cell control. This reduction is resulted from a non-specific reaction of those SiNCs to the cells.

After the confirmation of the PD-L1 knockdown on both mRNA and the protein levels, the effects of the PD-L1 knockdown in terms of cell functions and survival were also studied. It is well known that a variety of biomarkers, including CD25, CD69, CD71, CD95, CD137, CD147, OX40, ICOS, and HLA-DR, are expressed on the surface of activated T cells. Moreover, these activated T cells release specific cytokines such as interleukin-2 (IL-2) and interferon-*γ* (IFN-*γ*), in order to subsequently induce an immune reaction [21]. Here, we tracked the T-cell activation markers—CD25, CD69, and CD71 (Figure 4c), and cell proliferation (Figure 4d) on day 2, after the siRNA delivery. CD8^+^ T-cells treated with both SiNCs containing the target siRNA, showed an induction of all activation markers’ expression and an increase of cell proliferation, compared to the control sample. Furthermore, IFN-*γ*, TNF-*β*, and IL-2 secretion of the treated T-cells were also evaluated on day 2 (Figure 4e–g). The SiNCs containing the target siRNAs-treated cells produced significantly more of these cytokines than the cell control, especially IL-2 of the T-cells that were treated with the SiNCs containing target siRNA#6, except for the SiNCs containing target siRNA#3. These results suggest that even PD-L1 was partially knocked down in the CD8^+^ T-cells treated with SiNCs containing the target siRNA. But the effects of PD-L1 knockdown were successfully obtained. In order to prolong gene silencing, an increase of siRNA stability [22] or re-treatment with the siRNA-nanocarrier system could be considered as options.

## 4. Discussion

It is generally accepted in the research field of immune checkpoint proteins that PD-1 on the T-cell surface is responsible for the negative regulation of activated T-cells via binding, as its ligands PD-L1 and PD-L2 on dendritic cell surfaces are the cause of T-cell inactivation [12]. This inhibition interaction can occur between T-cell populations, given that PD-1 and PD-L1 are in fact expressed on activated T-cell surfaces [13]. Thus, the immune checkpoint inhibitors that target both PD-1 and PD-L1 could help restore the T-cells’ ability to attack cancer cells [23]. However, further investigation on destroying the activity of the improved T-cells function, restored from this siRNA-mediated gene silencing system, in the fight against cancerous cells, still needs to be undertaken, in order to prove the efficiency for use in T-cell immunotherapy for the treatment of cancer. In this study, we use SiNC as a suitable nanocarrier that can deliver siRNA to CD8^+^ T-cells. The siRNA was released and function was proved by the decrease of target mRNA and protein expression. The silencing of PD-L1 could not only enhance the activation markers’ expression, but also promote cell proliferation, and cytokine secretion, which is a promising strategy to improve T-cell function.

## 5. Conclusions

These results indicate that the silica nanocapsules were able to deliver the siRNA target to the CD8^+^ T-cells with subsequent release and knockdown of PD-L1. This was shown for both, mRNA and protein levels, and as a result, enhanced the T-cell survival and functions. This NC-LUT exhibited an excellent potential for use as a nanocarrier in the biomolecule delivery to hard-to-transfect T-cells.

## Figures and Tables

**Table 1:** Overview of silica nanocapsules encapsulated Oligo Cy5.

Sample	Diameter (nm)	Zeta Potential (mV)	Encapsulation Efficiency of Oligo Cy5
NC1-Oligo Cy5-CTAC	237 ± 104	15	39%
NC1-Oligo Cy5-LUT	262 ± 100	−10	21%
NC2-Oligo Cy5-CTAC	231 ± 111	5	63%
NC2-Oligo Cy5-LUT	248 ± 108	−8	44%

## Supplementary Materials

Supporting Information is available online at https://www.mdpi.com/2073-4409/9/9/2043/s1. Figure S1: TEM micrographs of SiNCs.
