# Supplementary figures and images for: Cellular Uptake of siRNA-Loaded Nanocarriers to Knockdown PD-L1: Strategies to Improve T-cell Functions
Source: Cells. 2020 Sep 7;9(9):2043. doi: 10.3390/cells9092043 (PMC7565787; doi:10.3390/cells9092043)

## Supporting Information

**NC1**

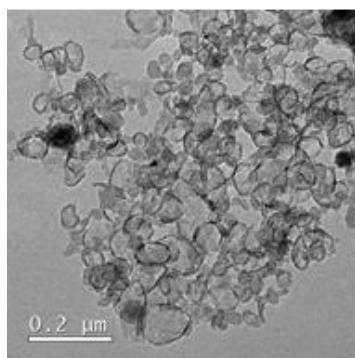

**NC2**

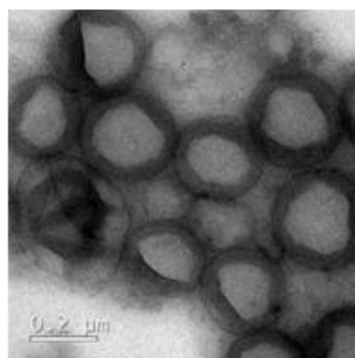

**Oligo Cy5-CTAC**

**Figure S1.** TEM micrographs of SiNCs.

Supplement: Supplementary file 1 [file cells-09-02043-s001.pdf]
